# The L1-type cell adhesion molecule Neuroglian is necessary for maintenance of sensory axon advance in the *Drosophila *embryo

**DOI:** 10.1186/1749-8104-3-10

**Published:** 2008-04-08

**Authors:** Veronica Martin, Eli Mrkusich, Martin C Steinel, Jason Rice, David J Merritt, Paul M Whitington

**Affiliations:** 1 Department of Anatomy and Cell Biology, University of Melbourne, VIC 3010, Australia; 2 School of Integrative Biology, University of Queensland, Brisbane, QLD 4072, Australia

## Abstract

**Background:**

Cell adhesion molecules have long been implicated in the regulation of axon growth, but the precise cellular roles played by individual cell adhesion molecules and the molecular basis for their action are still not well understood. We have used the sensory system of the *Drosophila *embryo to shed light on the mechanism by which the L1-type cell adhesion molecule Neuroglian regulates axon growth.

**Results:**

We have found a highly penetrant sensory axon stalling phenotype in *neuroglian *mutant embryos. Axons stalled at a variety of positions along their normal trajectory, but most commonly in the periphery some distance along the peripheral nerve. All lateral and dorsal cluster sensory neurons examined, except for the dorsal cluster neuron dbd, showed stalling. Sensory axons were never seen to project along inappropriate pathways in *neuroglian *mutants and stalled axons showed normal patterns of fasciculation within nerves. The growth cones of stalled axons possessed a simple morphology, similar to their appearance in wild-type embryos when advancing along nerves. Driving expression of the wild-type form of Neuroglian in sensory neurons alone rescued the *neuroglian *mutant phenotype of both pioneering and follower neurons. A partial rescue was achieved by expressing the Neuroglian extracellular domain. Over/mis-expression of Neuroglian in all neurons, oenocytes or trachea had no apparent effect on sensory axon growth.

**Conclusion:**

We conclude that Neuroglian is necessary to maintain axon advance along axonal substrates, but is not required for initiation of axon outgrowth, axon fasciculation or recognition of correct growth substrates. Expression of Neuroglian in sensory neurons alone is sufficient to promote axon advance and the intracellular region of the molecule is largely dispensable for this function. It is unlikely, therefore, that Nrg acts as a molecular 'clutch' to couple adhesion of F-actin within the growth cone to the extracellular substrate. Rather, we suggest that Neuroglian mediates sensory axon advance by promoting adhesion of the surface of the growth cone to its substrate. Our finding that stalling of a pioneer sensory neuron is rescued by driving Neuroglian in sensory neurons alone may suggest that Neuroglian can act in a heterophilic fashion.

## Background

The growing axon is directed to its synaptic target by interactions between molecules on the surface of the growth cone and specific cues in its environment. Such molecular interactions either promote or inhibit the advance of the growth cone in a particular direction. A key element in models that seek to explain axon advance is the level of adhesion between the growth cone and its substrate. A large body of experimental data from both *in vitro *and *in vivo *systems points to the important role that cell adhesion molecules (CAMs) play in the regulation of axon advance, fasciculation and turning (reviewed in [1-3]).

One of the best studied classes of CAMs is the L1-type family, represented in vertebrates by the four members L1, Neurofascin, Nr-CAM and CHL1. Invertebrate homologues of L1 proteins include the insect protein Neuroglian (Nrg) [4] and the leech protein Tractin [5]. These molecules all share an extracellular region of six immunoglobulin-like domains, three to five Fibronectin type III domains and a single transmembrane domain. The cytoplasmic region contains ankyrin (FIGQY) and Ezrin-Radixin-Moesin (ERM) binding motifs, which allow the molecule to link to spectrin and actin, respectively. Most members of the L1-type family mediate cell-cell adhesion *in vitro *by a homophilic mechanism, although heterophilic interactions have also been reported for some vertebrate L1-type members (reviewed in [4]).

The genetic tools available in *Drosophila *have been particularly useful in elucidating the roles played by L1-type CAMs in the regulation of axon growth *in vivo *and the molecular mechanisms underlying those functions. The fly genome contains only one member of this family, *nrg*, which undergoes alternative splicing to generate two transcripts [6,7]. These encode a short form of the protein, Nrg^167^, which is expressed widely on epidermis, muscle, glia and trachea, and a longer form, Nrg^180^, which is restricted to neurons. The two isoforms have identical extracellular and transmembrane regions and differ only in the carboxy-terminal ends of their cytoplasmic domains.

Nrg has been shown to regulate axon growth in the peripheral nervous system (PNS) during both embryonic and post-embryonic development of *Drosophila*. Null *nrg *mutants display a highly penetrant motor axon stalling phenotype in the body wall of the embryo [7]. This phenotype, together with the normal Nrg expression pattern, suggests that Nrg mediates the recognition of target muscles by embryonic motor axons by a homophilic mechanism, although this hypothesis has not yet been formally tested.

Nrg is also involved in guidance of bristle mechanosensory (BM) and ocellar pioneer (OP) axons in the adult ocellar sensory system (OSS) of *Drosophila *[8,9]. Ablation of Nrg function specifically in late larval stages with a temperature sensitive loss-of-function (LOF) allele results in several types of axon defects. These include abnormal growth of OP axons along the head epidermis, stalling or premature detachment of BM axons from the epidermis and failure of both OP and BM axons to project to the brain. The complexity of these mutant phenotypes suggests that Nrg plays multiple roles in axon guidance in this system, including promotion of axon advance, axon fasciculation and axon turning. However, the precise nature of those roles remains to be clarified. It is also unclear whether Nrg acts purely as a cell-cell adhesion molecule in this context and whether its effects are mediated by homophilic or heterophilic molecular interactions.

There are conflicting data as to whether the intracellular domain of Nrg is necessary to mediate the effects of this molecule on axon growth. Expressing an artificial, glycosylphosphatidylinisotol (GPI)-anchored form of Nrg in wing sensory neurons and their epidermal substrate can generate the same gain-of-function phenotypes as the full length protein [10], suggesting that only the extracellular portion of Nrg is essential for its function in this system. On the other hand, Nrg^167^, which has an identical extracellular region to Nrg^180^, fails to rescue OSS axon defects in the *nrg *LOF mutant [9]. This result suggests that the intracellular Nrg region does play an important role in the regulation of axon growth.

Clearly, many questions remain concerning the mechanism of action of Nrg in the regulation of axon growth. We have chosen the sensory nervous system of the *Drosophila *embryo as a model to further explore these issues. The cellular events underlying the peripheral growth of individual sensory axons are well characterized [11,12] and several of the molecules involved in their guidance have been identified [13-15], providing us with an ideal opportunity to link molecular mechanisms of Nrg function to cellular outcomes. Studying the embryonic sensory system means that we can examine phenotypes in embryonic lethal, null *nrg *mutants, rather than studying adult phenotypes of hypomorphic or temperature sensitive mutants. This should provide us with a more reliable assessment of normal gene function. Our analysis shows that Nrg is essential for maintenance of sensory axon advance in the embryo, but not for correct axon fasciculation or pathfinding. The requirement for Neuroglian function in embryonic sensory neurons appears, therefore, to be less complex than in other contexts in *Drosophila*. Nrg expression is required in sensory neurons alone and the extracellular region of the molecule is at least partially able to perform this function. Our findings may also suggest that Nrg can promote sensory axon advance by a mechanism involving heterophilic molecular interactions.

## Results

### Nrg is required for maintenance of sensory axon advance

**Figure 1:**
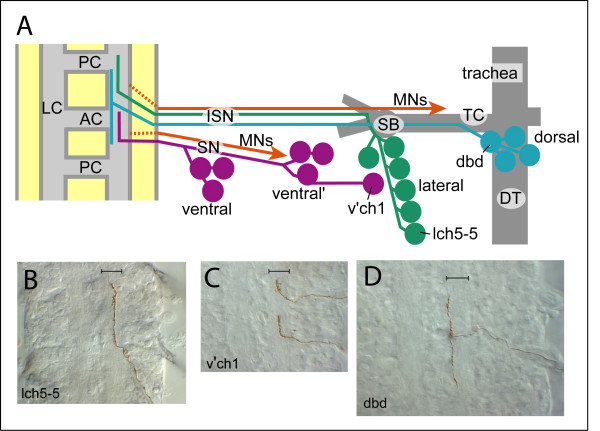
Axon morphology of sensory neurons in wild-type *Drosophila *embryo. **(a) **Diagram of the pathways and substrates followed by sensory axons in the *Drosophila *embryo. Only a subset of the sensory neurons in each of the dorsal (blue), lateral (green), ventral' and ventral clusters (purple) has been represented. The relative positions of the lch5-5, dbd and v'ch1 neurons are shown, together with their schematic axonal projections in the CNS. Major axon tracts in the CNS, the anterior commissure (AC), the posterior commissure (PC) and longitudinal connectives (LC) are shaded light grey. Motor axons (MNs, orange) originate within the CNS (dashed lines) and project into the periphery within the segmental (SN) or intersegmental (ISN) nerves. Tracheal branches, including the dorsal trunk (DT), transverse commissure (TC) and spiracular branch (SB) are shown in grey. **(b-d) **Central projections of lch5-5 (b), v'ch1 (c; two neurons in adjacent hemisegments filled) and dbd (d) axons in early stage 17 wild-type embryos, revealed by juxtacellular DiI labeling. All preparations were photo-converted, yielding a brown diaminobenzidine reaction product in the sensory axon and imaged with DIC optics to reveal axon tracts in the CNS. The bar in each diagram shows the medial-lateral extent of the LC, which is approximately 10 μm wide at this stage. lch5-5 and v'ch1 both project anteriorly in the middle of the LC, while the dbd axon has a bifurcating terminal projection located at the medial edge of the LC.

In this study, we have focused on the growth of axons from the dorsal and lateral sensory neuron clusters in abdominal segments A1 to A7. During normal development, sensory axons contact other sensory neurons or their axons shortly after emerging from the neuron cell body. The dorsal cluster axon fascicle initially grows internally to the transverse connective branch of the trachea, turns ventrally, joins the intersegmental nerve (ISN) in the dorsal body wall region and continues growing ventrally towards the central nervous system (CNS) [11,14]. It has previously been reported that the dorsal branch of the ISN is pioneered by the dorsal hair es neurons desA2 [11,16]. However, we find that when dbd and other dorsal cluster neurons are labeled with DiI in different hemisegments of the same embryo, dbd's axon has always advanced further along the ISN than other axons (Steinel and Whitington, unpublished observations). Lateral cluster axons fasciculate as they initially grow anteriorly, following the pioneering axon for the lateral cluster, lch5-1. They then turn internally along the spiracular branch of the trachea, fasciculate with efferent motor axons of the ISN and advance ventrally towards the CNS parallel to, but separated from, the dorsal cluster sensory fascicle [12]. While v'ch1 lies in the lateral cluster, its axon follows a different path to the rest of these neurons: it initially grows ventrally to contact cell bodies of sensory neurons in the v' (ventral') cluster and follows their axon fascicle towards the CNS within the segmental nerve (SN) [15]. SN axons converge with ISN axons at the edge of the ventral nerve cord. The sensory axonal pathways are summarized in schematic form in Figure 1a. After entering the CNS, sensory axons advance into the neuropile. The lateral cluster lch5 axons pause at this stage (early stage 16) and acquire a flattened growth cone morphology with multiple filopodia, before turning antero-posteriorly along the longitudinal connectives (LC). Sensory neurons of different modalities and from different positions in the body wall diverge and form specific central arborization patterns within the neuropile. These specific sensory projections have been established by stages 16–17, depending on the neuron type [17]. Figure 1b–d shows the mature embryonic projections of the lateral cluster neuron lch5-5, the v'ch1 neuron and the dorsal cluster neuron dbd, as revealed by single neuron, juxtacellular staining with DiI.

**Figure 2:**
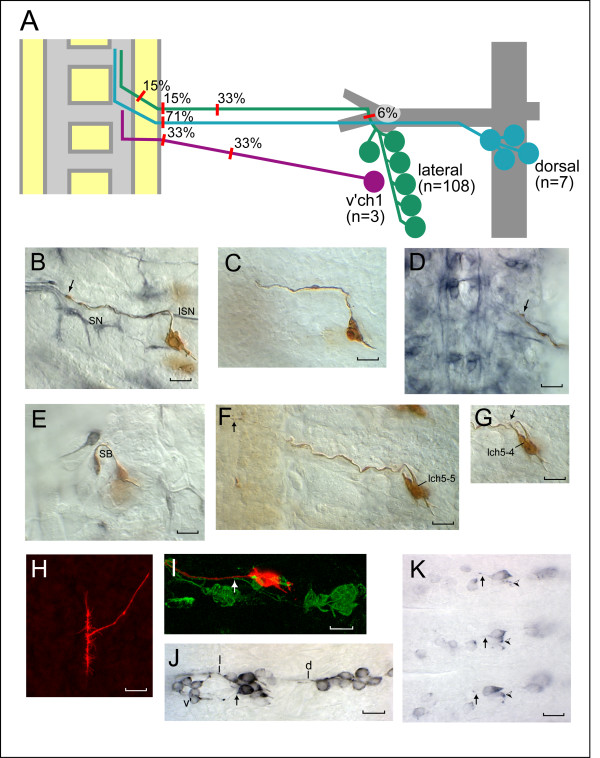
Sensory axon defects in *nrg *LOF mutants. **(a) **Summary of sensory axon stalling positions (indicated by red lines) and percentage of neurons that stalled at those positions in *nrg *LOF mutant embryos (*nrg*^14^*/Y *and *nrg*^17^*/Y *data pooled). Data on the green line are for all lateral cluster neurons combined (n = 108), on the blue line for all dorsal cluster neurons combined (n = 7), excluding dbd, and on the purple line for v'ch1 alone (n = 3). **(b-i) **Axon morphology of sensory neurons in *nrg*^14^*/Y *embryos as revealed by single neuron DiI labeling. (b-g) DIC images of photoconverted preparations; (h, i) confocal z-projections. Embryos in (b, d, e) were co-immunostained with mAb1D4 to reveal motor axon projections in the periphery, while the embryo in (i) was co-stained with mAb22C10 to reveal all other sensory axons. (b) An lch5-5 stall in the ISN, before its junction with the SN. The arrow shows the position of the lch5-5 growth cone. (c) An lch5-5 stall in the ISN at a similar position to (b). (d) An lch5-5 stall in the ISN nerve root within the CNS. The arrow indicates the position of the stall. (e) An lch5-3 stall just before entry into the ISN. The axon has grown internally along the spiracular branch (SB). (f) An lch5-5 axon has stalled at the edge of the LC in the CNS (arrow). (g) The adjacent lch5-4 in the same hemisegment as the lch5-5 neuron in (f) has stalled a short distance from the cell body (arrow). (f, g) also show the disruption in the alignment of the lch5 cell bodies often seen in the *nrg *mutant. (h) dbd shows a normal, bifurcating axon projection within the CNS in the *nrg *mutant. (i) The axon of lch5-1 (red) lies separate to the dorsal cluster axons (arrow, green, 0.5 μm separation) within the ISN in this stage 16 *nrg *mutant embryo. **(j, k) ***nrg*^14^*/Y *embryos immuno-stained with mAb22C10. (j) Segment A3 in a stage 15 embryo showing a normal distribution of sensory nerves. This montage shows the initial segment of the dorsal cluster axon fascicle (d) and the lateral cluster fascicle to the point where it turns internally to join the ISN (l). The axon of v'ch1 (arrow) follows a normal course to the v' cluster (v'). (k) Segments A3-5 in a stage 13 embryo. lch5-1 has projected a short axon in all three segments (arrow), while the soma of lch5-2 is only weakly stained and has yet to form an axon (arrowhead). Scale bar = 10 μm in all figures.

We visualized sensory axon morphology in mid-stage 16 to stage 17 (gastric caecae length 50–200 μm) *nrg *LOF mutant embryos by DiI labeling. While there is some normal variability in the timing of axon growth, all of the identified sensory neurons we examined have entered the neuropile and begun to form terminal arborizations by mid-stage 16 in 100% of wild-type embryos (n = 19 for lch5-1, 27 for lch5-5, 19 for v'ch1, and 8 for dbd). Sensory neurons showed a highly penetrant axon stalling defect in *nrg *mutant embryos. Axons were judged to have stalled if they had not entered the neuropile. Stalled axons consistently showed a simple morphology, terminating in the nerve with a simple, club-shaped growth cone with, at most, one or two projecting filopodia (Figure 2b–g). Cell body and dendrite morphology of sensory neurons were relatively normal, although, as previously reported [7], the regular alignment of lch5 cell bodies was often disrupted in *nrg *mutant embryos (Figure 2f,g).

Axon stalling occurred at a variety of positions along the normal axon trajectory. Axons seldom stalled before entry into the ISN: Figure 2e shows an example of such an early lch5-3 stall. Most lateral cluster axon stalls occurred in the periphery some distance along the peripheral nerve (Figure 2a–c), but in a number of cases axons stalled just before they entered the CNS or shortly after entry (Figure 2a,d). Dorsal cluster axon stalls occurred most frequently just before entry to the CNS. The location of axon stalls did not obviously correlate with the position of nerve branches or other structural features. The inherent variability in the stalling point is highlighted by the observation that adjacent neurons of the same lch5 cluster can stall at very different points along the nerve (Figure 2f,g).

We injected a range of identified lateral and dorsal cluster sensory neurons, including the five lateral chordotonal neurons lch5-1 to lch5-5, v'ch1, lesB, ldaA, ldaB, dbd as well as other cells in the lateral and dorsal clusters that could not be unambiguously identified at the individual neuron level. Axon stalling was seen for all of the neurons examined, except for dbd. The overall stalling frequency for lch5-5 and for all types of neurons combined, excluding dbd, was the same for the *nrg*^14 ^and *nrg*^17 ^LOF alleles (Table 1). We collected sufficient data for two neurons, lch5-1 and lch5-5, to enable us to compare their stalling rates: lch5-1 stalled less frequently than lch5-5 (*P *< 0.01) in *nrg*^14^*/Y *embryos (Table 1).

**Table 1:** Axon stalling rates in mutant and transgenic *nrg *embryos

	Percentage of stalled axons (total n)*			
	
	Neuron			
		
Genotype	lch5-1	lch5-5	dbd	All neurons combined (excluding dbd)
*w*^1118^	0% (19)	0% (27)	0% (8)	-
*nrg*^14^*/Y*	48%^† ^(21)	84%^† ^(25)	0% (14)	70% (87)
*nrg*^17^*/Y*	-	79% (28)	-	71% (45)
*nrg*^14^*/Y;P0163-GAL4/UAS-nrg*^180^	0% (22)	20%^‡ ^(20)	-	-
*nrg*^14^*/Y;UAS-nrg*^*GPI*^*;P0163-GAL4*	-	48%^‡ ^(25)	-	-
*P0163-GAL4/UAS-nrg*^180^	-	0% (31)	-	-
*elav-GAL4/UAS-nrg*^180^	-	0% (30)	-	-
*P0163-GAL4/UAS-nrg*^*GPI*^	-	7% (27)	-	-
*elav-GAL4/UAS-nrg*^*GPI*^	-	6% (16)	-	-
*sal-GAL4;UAS-nrg*^180^	-	0% (35)	-	
*btl-GAL4/UAS-nrg*^180^	-	0% (23)	-	-

*Based on morphology of DiI injected neurons in mid-stage 16 to stage 17 embryos. Axons were judged to have stalled if they had failed to reach the CNS neuropile. See Figure 2a for locations of stalling points for different types of neurons. ^†^These values are significantly different to each other (*P *< 0.01). ^‡^These values are significantly different to the *nrg*^14^*/Y *stalling rates (*P *< 0.01) and to each other (*P *= 0.05).

The dorsal cluster neuron dbd appears to be unaffected by loss of *nrg *function. In all 14 dbd neurons filled with DiI, the axon showed the bifurcating projection in the CNS, typical of a wild-type neuron at this stage (compare Figures 1d and 2h; Table 1).

Sensory axons were never seen to project along inappropriate pathways in *nrg *mutants. This was established by injecting single sensory neurons with DiI, then immuno-staining the embryo with mAb1D4 or mAb22C10, which reveals the morphology of all motor axons and sensory axons, respectively. Stalled sensory axons in the mutant showed the same close association with ISN motor axons and separation from dorsal sensory axons (0.5–1.0 μm) as seen in wild-type embryos (Figure 2b,d,i). The overall distribution of sensory nerves was normal in *nrg *mutants (Figure 2j). This result provides further support for our conclusion that sensory axons fasciculate normally in *nrg *mutants. Sensory axon stalling defects were not evident in embryos stained with mAb22C10 alone (as has been noted previously [7]), presumably because individual sensory axons stall at different, variable locations rather than consistently at particular sites along the nerve. The relative timing of axon outgrowth from lateral cluster neurons, as assayed by mAb22C10 immuno-staining, is also unaffected by loss of *nrg *function: as in wild-type embryos, lch5-1 is the pioneer for the lateral cluster fascicle in *nrg*^14 ^mutants (in 24/24 hemisegments, Figure 2k).

### Expression of Nrg in sensory neurons is sufficient to rescue the *nrg *LOF phenotype

During embryogenesis, Nrg shows a widespread pattern of expression. Of particular relevance to its role in the regulation of sensory axon growth is the expression of Nrg^180 ^on sensory neuron cell bodies and sensory and motor axons as well as the expression of Nrg^167 ^on glial cells and trachea [6,18]. Nrg could, therefore, mediate sensory axon extension through its expression in sensory axons or one or more of these various cell types, all of which have been implicated as possible growth substrates in sensory axon guidance.

**Figure 3:**
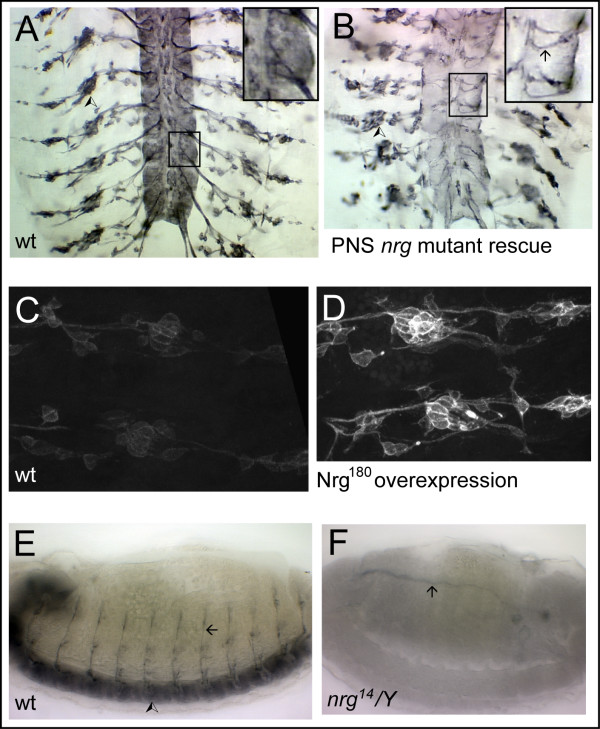
Expression of Nrg in **(a, c, e) **wild-type, **(b) **mutant rescue, **(d) **over-expression and **(f) **mutant embryos. All embryos were immuno-stained with mAb BP104, which recognizes an epitope on Nrg^180^. HRP-conjugated secondary antibody and bright field illumination was used in (a, b, e, f) and Alexa488-conjugated secondary antibody and confocal microscopy in (c, d). (a, b) Dorsal views of filleted stage 16 embryos. (a) In the wild-type embryo, Nrg^180 ^is expressed by many central neurons, as well as in sensory neuron cell bodies (arrowhead shows lateral cluster) and their axons. Inset shows a higher magnification view of the area in the rectangle. (b) In an *nrg*^14^*/Y;P0163-GAL4/UAS-nrg*^18 ^embryo, Nrg^180 ^is expressed in sensory neurons (arrowhead shows lateral cluster) and their axons, but not in central neurons. Stained structures in the CNS are sensory axons, shown at higher magnification in the inset (arrow). Note the lack of staining of cell bodies of neurons in the CNS. (c, d) Lateral view of PNS in two adjacent hemisegments in wild-type (c) and *P0163-GAL4/UAS-nrg*^18 ^(d) stage 17 embryos. The same confocal microscope settings were used when imaging both embryos. The stronger staining in the embryo in (d) shows that the *P0163-GAL4 *driver was effective in driving elevated levels of Nrg^180 ^expression in sensory neurons. (e, f) Lateral view of stage 16 wild-type (e) and *nrg*^14^*/Y *mutant (f) embryos. In contrast to the positive staining of sensory axons (arrow) and the CNS (arrowhead) in the wild-type embryo, no staining is evident in these structures in the mutant embryo. Weak staining of the lumen of the dorsal trunk of the trachea (arrow), likely to be non-specific, is evident.

To determine whether Nrg expression in sensory neurons is sufficient to promote sensory axon extension, we drove expression of the wild-type form of Nrg^180 ^using the *P0163-GAL4 *driver in the *nrg*^14 ^null mutant background. We focused on the two neurons for which we had multiple fills in *nrg*^14 ^mutant embryos – lch5-1 and lch5-5. To confirm that expression of Nrg^180 ^had been specifically restored in sensory neurons, we carried out BP104 immunohistochemistry (which recognizes an epitope specific to Nrg^180^) on *nrg*^14^*/Y;P0163-GAL4/UAS-nrg*^180 ^embryos. Positive staining was seen in sensory neurons, including the lch5s and their axons, but not in central neurons (compare Figure 3a and 3b).

Embryos of this genotype were selected and lch5-1 or lch5-5 neurons were injected with DiI. The rate of lch5-5 axon stalling was significantly lower frequency (*P *< 0.001) than in the *nrg*^14 ^mutant (Table 1). None of the axons that stalled did so in the PNS, whereas the majority (57%) of the stalls in the *nrg*^14 ^mutant occurred in the PNS.

We injected 22 lch5-1 neurons in *nrg*^14^*/Y;P0163-GAL4/UAS-nrg*^180 ^embryos. None of these axons had stalled: all had either turned rostro-caudally or had an expanded growth cone located in a normal ventro-medial position in the LC (Table 1).

We conclude that Nrg expression in sensory neurons is sufficient to mediate its effects on both lch5-1 and lch5-5 axon extension.

### The extracellular region of Nrg is sufficient to partially rescue the sensory axon stall phenotype

To test whether the extracellular region alone can mediate the effects of Nrg on sensory axon growth, we attempted to rescue the *nrg *mutant phenotype by driving expression of a GPI-anchored form of the Nrg extracellular domain [19] in sensory neurons using the *P0163-GAL4 *driver.

We injected a total of 25 lch5-5 neurons with DiI in *nrg*^14^*/Y;UAS-nrg*^*GPI*^*;P0163-GAL4 *embryos. The axon stalling rate in these embryos was significantly lower (*P *< 0.01) than for the *nrg*^14 ^mutant (Table 1). Furthermore, only 2 of the 12 axons that did stall did so in the PNS. These results show that the extracellular region of Nrg can at least partially rescue the mutant phenotype.

The *UAS-nrg*^*GPI *^stalling rate is significantly higher than the *UAS-nrg*^180 ^rate (albeit only at a low significance level, *P *= 0.05). This may suggest that the *nrg*^*GPI *^construct is not as effective in promoting axon advance as the full length *nrg *construct, although it is also possible that differences in the level of expression of the two constructs could account for this apparent difference in rescuing ability.

### Over-expression of Nrg has no effect on sensory axon growth

To determine whether, as in the adult *Drosophila *wing [10], overexpression of Nrg results in sensory axon defects, we over-expressed Nrg^180 ^in the embryonic PNS using either the *P0163-GAL4 *driver or the pan-neural *elav-GAL4 *driver. Confocal microscope observations on wild-type and *P0163-GAL4 X UAS-nrg*^180 ^embryos stained with BP104 under the same conditions confirmed that Nrg^180 ^was expressed at higher levels in the transgenic embryos than in wild-type embryos (Figure 3c,d).

Neither form of overexpression resulted in sensory axon stalling as determined by DiI injections (n = 31 lch5-5 neurons in *P0163-GAL4 X UAS-nrg*^180 ^embryos, n = 30 lch5-5 neurons in *elav-GAL4 X UAS-nrg*^180 ^embryos; Table 1). In one *P0163-GAL4 X UAS-nrg*^180 ^embryo, the lch5-5 axon bifurcated in the PNS, but both branches grew into and arborized within the CNS. Furthermore, no axonal misprojections were evident in mAb22C10 stained embryos (n = 98 hemisegments in *P0163-GAL4 X UAS-nrg*^180 ^embryos, n = 91 hemisegments in *elav-GAL4/UAS-nrg*^180 ^embryos). mAb49c4 immunohistochemistry, which labels chordotonal neurons specifically, did reveal an aberrant dorsal misprojection of lch5 axons but only in 1/85 hemisegments of *elav-GAL4 X UAS-nrg*^180 ^embryos.

We also overexpressed the GPI-anchored form of Nrg using the *P0163-GAL4 *or *elav-GAL4 *drivers. Of 27 lch5-5 neurons labeled with DiI in *P0163-GAL4 X UAS-nrg*^*GPI *^embryos, 2 showed axon stalling in the PNS, while the other 25 had a normal axon projection in the CNS (Table 1). Only one lch5-5 axon stall was seen in 16 *elav-GAL4 X UAS-nrg*^*GPI *^embryos (Table 1). Thus, unlike adult wing sensory neurons [10], expression of Nrg^*GPI *^in embryonic sensory neurons has only a weak effect on their axon growth.

Misexpression of Nrg^180 ^in the oenocytes (with the *sal-GAL4 *driver) or the trachea (with the *btl-GAL4 *driver) – cell types that lie in the vicinity of the lch5 neurons and their axons [12,15] – has no apparent effect on sensory axon growth (Table 1).

## Discussion

We have used the embryonic sensory system of the *Drosophila *embryo to determine the consequences of complete ablation of Nrg function on sensory axon growth. *nrg *LOF mutants show a highly penetrant sensory axon stalling phenotype. This defect is displayed by all neurons examined, except for the dorsal cluster neuron, dbd, which apparently leads other dorsal cluster axons as it grows along the ISN into the CNS. The fact that this pioneering neuron is unaffected by loss of *nrg *function may suggest that Nrg is necessary for axon advance only along axonal substrates. This idea is supported by our observation that lateral cluster axons in *nrg *mutants almost always advance successfully along their initial non-neuronal growth substrate, the spiracular branch of the trachea, and stall only after they have subsequently joined the ISN.

As sensory axons in *nrg *mutants generally advance for some distance along nerves before stalling, Nrg is apparently required to maintain axon growth along axonal substrates, rather than to initiate it. Most axon stalls occur in the periphery, but their locations are quite variable and do not obviously coincide with particular structures, such as nerve branches, pointing to a stochastic component in the stalling process.

Stalled sensory axons in *nrg *mutants are always found in their correct nerve pathways. The absence of axon misprojection phenotypes shows that Nrg is not necessary for recognition of correct growth substrates by sensory axons.

We also observed that sensory axons in *nrg *mutants fasciculate normally with other axons. The simple growth cone morphology of the stalled axons – club-shaped with few filopodia – is similar to that seen in wild-type sensory axons when they are growing along nerves. These observations show that Nrg is not necessary for axon-axon fasciculation in this system. The Ig-CAM Fasciclin2 (Fas2) is a key regulator of motor axon fasciculation in the *Drosophila *embryo [20] and also acts redundantly to Nrg in the regulation of OP axon growth in the adult OSS [9]. However, Fas2 is unlikely to be acting redundantly with Nrg in regulating sensory axon fasciculation in the embryo, as embryonic sensory axons do not express Fas2 [18]. In addition, the pattern of 22C10 sensory nerve staining is normal in *fas2 *LOF mutants and in embryos over/misexpressing Fas2 in sensory neurons and their growth substrates (Steinel and Whitington, unpublished observations). Whether Nrg acts redundantly with some other CAM to mediate sensory axon fasciculation remains to be determined.

The sensory axon defects seen in *nrg *mutant embryos are similar to those reported for motor axons, although motor axons stall at a later stage of growth, close to their synaptic targets, the body wall muscles, and unlike sensory axons, some motor axons misproject in *nrg *mutants [7]. The *nrg *LOF phenotype in the embryonic sensory system is much more stereotypic than in the adult OSS [8]. This difference may indicate that Nrg performs more diverse functions in the adult OSS, including regulation of axon fasciculation and guidance in addition to axon advance.

While Nrg is expressed in both sensory axons and several of their growth substrates, including trachea and motor axons, our mutant rescue experiments demonstrate that expression in sensory axons alone is sufficient to mediate their advance. It is unlikely, therefore, that the sensory axon defects are secondary to other known morphological defects in *nrg *mutants, such as motor axon stalling [7] or failure of glial cell ensheathment of peripheral nerves [18].

An important, unresolved question is whether the effects of Nrg on axon growth are mediated by homophilic interactions between Nrg on the growth cone and its substrate or whether heterophilic interactions are involved. Substrate-bound Nrg has been shown to promote neurite extension from Nrg-expressing neurons *in vitro *[21], but it is unclear whether the same holds true *in vivo*. We have shown that expression of Nrg in sensory neurons completely rescues the axon stalling phenotype of lch5-1 seen in *nrg *mutants. In wild-type embryos and in *nrg *mutants, lch5-1 is the pioneer for the lateral cluster fascicle and associates with motor axons, rather than dorsal cluster sensory axons as it grows towards the CNS. Our lch5-1 mutant rescue findings might suggest, therefore, that Nrg is acting in a heterophilic fashion to promote advance of this axon. This conclusion rests upon the assumption that, in *nrg *mutant embryos in which the lch5-1 stall phenotype has been rescued, the lch5-1 growth cone does not employ the dorsal sensory axons as a growth substrate as it advances along the ISN. Observations of the dynamics of lch5-1 growth cone activity, and its association with sensory and motor axons in the ISN as it advances along the nerve in these rescued embryos, would help to resolve this question.

Our mutant rescue experiments show that the intracellular region of Nrg is largely dispensable for its role in promoting sensory axon advance. It has long been known that Nrg-mediated cell-cell adhesion does not require the intracellular region of the molecule [19]. However, mutation of the intracellular ankyrin-binding domain of L1-type CAMs does have clear effects on their membrane mobility [22] and their coupling to retrograde actin flow in growth cones [23]. Given the linkage between ankyrins and the actin-spectrin cytoskeleton, this has led to suggestions that L1-type CAMs may transmit traction force generated by actin flow to the extracellular substrate, as posited by the 'molecular clutch' hypothesis [23]. The recent finding that mitogen-activated protein kinase phosphorylation of the ankyrin-binding site regulates neurite growth from cerebellar granule neurons on a Ng-CAM coated substrate [24] highlights the potential importance of the intracellular region of L1-type CAMs in mediating their effects on axon growth. However, this view is difficult to reconcile with our current results. One possible explanation is that the *in vitro *models used in most vertebrate studies to date do not accurately reflect the *in vivo *functions of L1-type CAMs. Alternatively, Nrg, the *Drosophila *L1 homologue, may function differently to vertebrate L1-type CAMs in promoting axon growth.

Our finding that the intracellular region of Nrg is not essential for its ability to promote sensory axon advance raises the question of how Nrg-mediated adhesion of the growth cone to its substrate is coupled to retrograde F-actin flow. Current models assume that such a coupling provides the motive force for growth cone advance. One possibility is that Nrg interacts in *cis *with another receptor or CAM, which in turn is coupled to the cytoskeleton of the growth cone. A genetic screen, using the sensory axon stalling phenotype described in our study as an assay, would be one way of identifying such a Nrg-interacting molecule.

## Conclusion

Taken together, our results suggest that Nrg may mediate sensory axon advance along nerves in the *Drosophila *embryo by promoting adhesion between the surface of the growth cone and its axonal substrates. This adhesive interaction is likely to involve a heterophilic interaction between Nrg on the growth cone and some other, as yet unidentified molecule, on the substrate. Intracellular signaling by the Nrg molecule does not appear to be essential for its function in this context. Our findings for Nrg are mirrored by the recent discovery that the adhesive, rather than the signaling activity, of another CAM, *Drosophila *N-cadherin, is essential for its role in target selection of photoreceptor afferents [25].

## Materials and methods

### *Drosophila *stocks

All stocks were raised on standard cornmeal and sugar medium. Two *nrg *mutant alleles were used, *nrg*^14 ^and *nrg*^17 ^(previously called *nrg*^1 ^and *nrg*^2^, respectively [7]). *nrg*^14 ^is a protein null and *nrg*^17 ^is a hypomorph that shows a markedly reduced expression of Nrg protein in embryonic tissues. Immuno-staining of *nrg*^14^*/Y *embryos with BP104 confirmed the absence of Nrg^180 ^protein in these embryos during the period of sensory axon outgrowth (Figure 3e,f). Two *UAS-nrg *lines were used in *nrg *over-expression and mutant rescue experiments: full-length neuronal Nrg^180 ^(*UAS-nrg*^180^) and an artificial Nrg isoform (*UAS-nrg*^*GPI*^), in which the extracellular region of Nrg is membrane-tethered with a GPI-moiety [26]. The following *GAL4 *lines were used to drive expression of these *UAS *lines: *P0163-GAL4*, which drives expression in all sensory neurons and their support cells [27]; the pan-neural *elav-GAL4 *line [28]; *sal-GAL4*, which drives expression in oenocyte precursors [29]; and *btl-GAL4*, which drives expression in trachea [30]. All mutant or transgenic stocks were maintained over green fluorescent protein- or *lacZ*-marked balancer chromosomes to enable genotyping of embryos by fluorescence microscopy or anti-β-galactosidase immunohistochemistry. Crosses were conducted at 25°C with the exception of UAS-GAL4 crosses, which were carried out at either 25°C or 29°C.

### Immunohistochemistry and neuron dye filling

Eggs were collected for 6 hours at 25°C then placed at 18°C for an additional 16 hours, or collected overnight at 25°C on apple juice-agar plates with yeast paste. Embryos were staged on the basis of gut morphology [31] and length of gastric caecae (see Figure 9 in [32]). Embryos were stained using standard immunohistochemical methods [33], with the exception that DiI-labeled embryos were treated individually on-slide. Primary antibodies used were mAb22C10 (mouse IgG, developed by S Benzer and obtained from the Developmental Studies Hybridoma Bank (DSHB) developed under the auspices of the NICHD and maintained by the University of Iowa, diluted 1:10 in phosphate-buffered saline with 0.1% Tween-20 (PBT), anti-Nrg^180^, BP104 [6] (mouse IgG, developed by C Goodman and obtained from DSHB, used at 1:4), 49C4 [34] (mouse IgM, gift from Y-N Jan, used at 1:10), 1D4 [35] (mouse IgG, obtained from DSHB, used at 1:5), anti-sex-lethal (mouse IgG, developed by P Schedl and obtained from DSHB, used at 1:20) and anti-β-galactosidase (mouse IgG, Promega, Madison, Wisconsin, USA used at 1:500). HRP or Alexa488-conjugated anti-mouse IgG, IgM or anti-rabbit IgG secondary antibodies were used at dilutions of 1:250 or 1:500 (obtained from Jackson Immunologicals, West Grove, Pennsylvania, USA).

Sensory neurons were individually stained in intact embryos by juxtacellular injection of DiI, as previously described [12,17]. Following dye injection, preparations were photo-converted in the presence of 0.2% diaminobenzidine to give a permanent dark reaction product, and in some cases immuno-stained with mAb1D4 to reveal the relationship between the injected neuron and motor axons. Stained embryos were mounted in 70% glycerol in phosphate-buffered saline and viewed with a Zeiss Axioskop. Digital images were captured with a Dage-MTI DC330 video camera and a Scion CG-7 frame grabber. Projections of in-focus images in multiple focal planes were made manually using Adobe Photoshop 7.0/8.0 software. DiI fills of the dorsal cluster neuron dbd and embryos stained with Alexa488-conjugated secondary antibodies were imaged with a Zeiss LSM 5 Pascal microscope. Z-projections of multiple focal planes were made using Zeiss LSM Image Browser software.

## Abbreviations

BM: Bristle mechanosensory; CAM: Cell adhesion molecule; CNS: Central nervous system; DSHB: Developmental Studies Hybridoma Bank; EGFR: Epidermal growth factor receptor; Fas2: Fasciclin2; GPI: Glycosylphosphatidylinisotol; ISN: Intersegmental nerve; LC: Longitudinal connectives; LOF: Loss-of-function; Nrg: Neuroglian; OP: Ocellar pioneer; OSS: Ocellar sensory system; PNS: Peripheral nervous system; SN: Segmental nerve; v': Ventral'.

## Competing interests

The authors declare that they have no competing interests.

## Authors' contributions

VM and EM jointly carried out neuron dye filling and phenotypic analysis for all neuron types, except dbd, in *nrg *mutants and mutant rescue experiments. VM and MCS jointly carried out phenotypic analysis of effects of Nrg overexpression on lch5 morphology. JR and DJM carried out phenotypic analysis of dbd morphology in *nrg *mutants. PMW conceived of the study, participated in its design and coordination and was largely responsible for writing the manuscript. All authors read and approved the final manuscript.

## References

[B1] Walsh FS, Doherty P (1997). Neural cell adhesion molecules of the immunoglobulin superfamily: role in axon growth and guidance. Annu Rev Cell Dev Biol.

[B2] Chiba A, Keshishian H (1996). Neuronal pathfinding and recognition – roles of cell adhesion molecules. Dev Biology.

[B3] Maness PF, Schachner M (2007). Neural recognition molecules of the immunoglobulin superfamily: signaling transducers of axon guidance and neuronal migration. Nat Neurosci.

[B4] Hortsch M (2000). Structural and functional evolution of the L1 family: are four adhesion molecules better than one?. Mol Cell Neurosci.

[B5] Huang Y, Jellies J, Johansen KM, Johansen J (1997). Differential glycosylation of tractin and LeechCAM, two novel Ig superfamily members, regulates neurite extension and fascicle formation. J Cell Biol.

[B6] Hortsch M, Bieber AJ, Patel NH, Goodman CS (1990). Differential splicing generates a nervous system-specific form of *Drosophila *neuroglian. Neuron.

[B7] Hall SG, Bieber AJ (1997). Mutations in the *Drosophila *Neuroglian cell adhesion molecule affect motor neuron pathfinding and peripheral nervous system patterning. J Neurobiol.

[B8] Garcia-Alonso L, Romani S, Jimenez F (2000). The EGF and FGF receptors mediate neuroglian function to control growth cone decisions during sensory axon guidance in *Drosophila*. Neuron.

[B9] Kristiansen LV, Velasquez E, Romani S, Baars S, Berezin V, Bock E, Hortsch M, Garcia-Alonso L (2005). Genetic analysis of an overlapping functional requirement for L1- and NCAM-type proteins during sensory axon guidance in Drosophila. Mol Cell Neurosci.

[B10] Islam R, Kristiansen LV, Romani S, Garcia-Alonso L, Hortsch M (2004). Activation of EGF receptor kinase by L1-mediated homophilic cell interactions. Mol Biol Cell.

[B11] Hartenstein V (1988). Development of *Drosophila *larval sensory organs: spatiotemporal pattern of sensory neurones, peripheral axonal pathways and sensilla differentiation. Development.

[B12] Harris KL, Whitington PM (2001). Pathfinding by sensory axons in *Drosophila*: substrates and choice points in early lch5 axon outgrowth. J Neurobiol.

[B13] Giniger E, Jan LY, Jan YN (1993). Specifying the path of the intersegmental nerve of the *Drosophila *embryo – a role for Delta and Notch. Development.

[B14] Parsons L, Harris KL, Turner K, Whitington PM (2003). *roundabout *gene family functions during sensory axon guidance in the *Drosophila *embryo are mediated by both Slit-dependent and Slit-independent mechanisms. Dev Biol.

[B15] Bates KE, Whitington PM (2007). Semaphorin 2a secreted by oenocytes signals through plexin B and plexin A to guide sensory axons in the *Drosophila *embryo. Dev Biol.

[B16] Younossi-Hartenstein A, Hartenstein V (1993). The role of the tracheae and musculature during pathfinding of *Drosophila *embryonic sensory axons. Dev Biol.

[B17] Merritt DJ, Whitington PM (1995). Central projections of sensory neurons in the *Drosophila *embryo correlate with sensory modality, soma position, and proneural gene function. J Neurosci.

[B18] Banerjee S, Pillai AM, Paik R, Li J, Bhat MA (2006). Axonal ensheathment and septate junction formation in the peripheral nervous system of *Drosophila*. J Neurosci.

[B19] Hortsch M, Wang YME, Marikar Y, Bieber AJ (1995). The cytoplasmic domain of the *Drosophila *cell adhesion molecule neuroglian is not essential for its homophilic adhesive properties in S2 cells. Journal of Biological Chemistry.

[B20] Lin DM, Fetter RD, Kopczynski C, Grenningloh G, Goodman CS (1994). Genetic analysis of Fasciclin II in *Drosophila*: defasciculation, refasciculation, and altered fasciculation. Neuron.

[B21] Forni JJ, Romani S, Doherty P, Tear G (2004). Neuroglian and FasciclinII can promote neurite outgrowth via the FGF receptor Heartless. Mol Cell Neurosci.

[B22] Gil OD, Sakurai T, Bradley AE, Fink MY, Cassella MR, Kuo JA, Felsenfeld DP (2003). Ankyrin binding mediates L1CAM interactions with static components of the cytoskeleton and inhibits retrograde movement of L1CAM on the cell surface. J Cell Biol.

[B23] Nishimura K, Yoshihara F, Tojima T, Ooashi N, Yoon W, Mikoshiba K, Bennett V, Kamiguchi H (2003). L1-dependent neuritogenesis involves ankyrinB that mediates L1-CAM coupling with retrograde actin flow. J Cell Biol.

[B24] Whittard JD, Sakurai T, Cassella MR, Gazdoiu M, Felsenfeld DP (2006). MAP kinase pathway-dependent phosphorylation of the L1-CAM ankyrin binding site regulates neuronal growth. Mol Biol Cell.

[B25] Yonekura S, Xu L, Ting CY, Lee CH (2007). Adhesive but not signaling activity of Drosophila N-cadherin is essential for target selection of photoreceptor afferents. Dev Biol.

[B26] Hortsch M, Homer D, Malhotra JD, Chang S, Frankel J, Jefford G, Dubreuil RR (1998). Structural requirements for outside-in and inside-out signaling by *Drosophila *neuroglian, a member of the L1 family of cell adhesion molecules. J Cell Biol.

[B27] Hummel T, Krukkert K, Roos J, Davis G, Klambt C (2000). *Drosophila *Futsch/22C10 is a MAP1B-like protein required for dendritic and axonal development. Neuron.

[B28] Luo LQ, Liao YJ, Jan LY, Jan YN (1994). Distinct morphogenetic functions of similar small GTPases: *Drosophila *Drac1 is involved in axonal outgrowth and myoblast fusion. Genes & Devel.

[B29] Boube M, Llimargas M, Casanova J (2000). Cross-regulatory interactions among tracheal genes support a co-operative model for the induction of tracheal fates in the *Drosophila *embryo. Mech Dev.

[B30] Shiga Y, Tanaka-Matakatsu M, Hayashi S (1996). A nuclear GFP/β-galactosidase fusion protein as a marker for morphogenesis in living *Drosophila*. Dev Growth & Different.

[B31] Campos-Ortega JA, Hartenstein V (1985). The Embryonic Development of Drosophila melanogaster.

[B32] Merritt DJ, Hawken A, Whitington PM (1993). The role of the *cut *gene in the specification of central projections by sensory axons in *Drosophila*. Neuron.

[B33] Patel NH (1994). Imaging neuronal subsets and other cell types in whole-mount *Drosophila *embryos and larvae using antibody probes. Methods Cell Biol.

[B34] Kolodziej PA, Jan LY, Jan YN (1995). Mutations that affect the length, fasciculation, or ventral orientation of specific sensory axons in the *Drosophila *embryo. Neuron.

[B35] Van Vactor D, Sink H, Fambrough D, Tsoo R, Goodman CS (1993). Genes that control neuromuscular specificity in *Drosophila*. Cell.

